# Pregnancy-related risk factors for sex cord-stromal tumours and germ cell tumours in parous women: a registry-based study

**DOI:** 10.1038/s41416-020-0849-z

**Published:** 2020-04-27

**Authors:** Camilla Sköld, Tone Bjørge, Anders Ekbom, Anders Engeland, Mika Gissler, Tom Grotmol, Laura Madanat-Harjuoja, Anne Gulbech Ording, Britton Trabert, Steinar Tretli, Rebecca Troisi, Henrik Toft Sørensen, Ingrid Glimelius

**Affiliations:** 1 Department of Immunology, Genetics and Pathology, Uppsala University, Uppsala, Sweden; 2 Department of Global Public Health and Primary Care, University of Bergen, Bergen, Norway; 3 Cancer Registry of Norway, Oslo, Norway; 4 Department of Medicine, Division of Clinical Epidemiology, Karolinska Institutet, Stockholm, Sweden; 5 Department of Chronic Diseases and Ageing, Norwegian Institute of Public Health, Bergen, Norway; 6 Finnish Institute for Health and Welfare (THL), Helsinki, Finland; 7 Department of Neurobiology, Care Sciences and Society, Karolinska Institutet, Stockholm, Sweden; 8 Finnish Cancer Registry, Helsinki, Finland; 9 Department of Pediatrics, University of Helsinki and Helsinki University Hospital, Helsinki, Finland; 10 Department of Clinical Epidemiology, Aarhus University Hospital, Aarhus, Denmark; 11 Division of Cancer Epidemiology and Genetics, National Cancer Institute, National Institutes of Health, Department of Health and Human Services, Bethesda, MD USA

**Keywords:** Cancer epidemiology, Ovarian cancer

## Abstract

**Background:**

Non-epithelial ovarian cancers are divided into sex cord-stromal tumours (SCSTs) and germ cell tumours (GCTs). Whereas parity and other pregnancy-related factors are protective for epithelial ovarian cancer, their associations with SCSTs and GCTs remains unclear.

**Methods:**

Using data from the medical birth registries from Denmark, Finland, Norway and Sweden, we compared all parous women with a diagnosis of SCSTs (*n* = 420) or GCTs (*n* = 345) 1970–2013 with up to 10 parous controls (SCSTs *n* = 4041; GCTs *n* = 2942) matched on the cases’ birth year and country. We used conditional logistic regression to estimate odds ratios (ORs) with 95% confidence intervals (CIs) of associations between pregnancy-related factors and SCSTs and GCTs.

**Results:**

The risk of SCSTs, but not GCTs, decreased with higher age at last birth [≥40 versus <25 years: OR 0.48 (95% CI 0.23–0.98)]. The risk of SCSTs (but not GCTs) also decreased with shorter time since last birth. Number of births, preterm birth, preeclampsia, and offspring size were not associated with risk of SCSTs or GCTs.

**Conclusions:**

We found a decreased risk of SCSTs with higher age at last birth and shorter time since last birth. The risk of SCSTs (but not GCTs) may be influenced by the woman’s reproductive history.

## Background

Non-epithelial ovarian cancers are rare tumours that are classified as either sex cord-stromal tumours (SCSTs) or germ cell tumours (GCTs), each with several subtypes. The biology and tumour development are poorly understood and rarely studied in SCSTs and GCTs. Both are located in the interior of the ovaries, and many of the subtypes produce hormones. SCSTs develop in women of all ages, with a peak incidence between 50 and 55 years.^1,2^ Although exceptionally rare, GCTs account for the majority of the ovarian malignancies among women <30 years and unlike SCSTs, incidence declines with age.^3^

Whereas increasing number of births and higher age at birth are protective for epithelial ovarian cancer,^4,5^ prior studies evaluating SCSTs or GCTs have been largely inconsistent with respect to the role of number of births and age at births on risk. These inconsistencies could be due to the small number of cases studied (typically less than 80).^6–9^ In the largest study published to date, including 330 SCSTs and 149 GCTs, Adami et al. reported an association between increasing number of births and decreased risk of SCSTs and a borderline association with decreased risk of GCTs.^10^ Intrauterine factors have been suggested to have an impact on risk of both SCSTs and GCTs in the mother^8,11^ as well as in the offspring.^12,13^ Hence, there is reason to further investigate the influence of other pregnancy-related factors that might reflect hormonal exposure during pregnancy and a women’s life, such as pregnancy length, age at births and offspring size.

We have performed a population-based case-control study with pooled data from four Nordic countries to allow an investigation of a large number of non-epithelial ovarian cancer cases. Our aim was to evaluate the risk of SCSTs and GCTs (in the mothers) in relation to pregnancy and birth characteristics. To have a homogenous study group with data on the factors of interest for this study, only parous women were included. By analysing the patterns of possible associations, we hope to increase our insight in the development of these rare tumours.

## Methods

### Outcomes and definition of cases/controls

This case-control study was based on linked data from the nationwide and population-based medical birth registries (MBRs) and cancer registries in a Nordic collaboration, and the methods used have been described recently.^5^ The MBRs contain information from antenatal, obstetric, and neonatal medical records and are based on mandatory reporting of all births since 1973 (Denmark), 1987 (Finland), 1967 (Norway), and 1973 (Sweden).^14–18^ The cancer registries are based on compulsory reporting of all incident tumours and cover the entire population since 1943 (Denmark), 1953 (Finland and Norway), and 1958 (Sweden). Case reporting is essentially complete and data validity high.^19–22^ The unique identifier assigned to each citizen in the Nordic countries at birth or upon immigration made accurate record linkages across the registries possible.

We included women recorded with a pregnancy lasting longer than 22 weeks in the MBRs who later developed malignant SCSTs and GCTs and were registered in a cancer registry between 1973 and 2011 in Denmark, 1987 and 2012 in Finland, 1967 and 2013 in Norway and 1973 and 2013 in Sweden (Table 1). Cases were free of other cancers at time of ovarian cancer diagnosis Ovarian cancer was defined by ICD-10/ICD-O-3 code C56.9, and by ICD-7 code 175.0 in Sweden before 1993, and ICD-O/2/ICD-O/3 codes and WHO/HS/CAN/C24.1 codes, as defined by the International Agency for Research on Cancer,^23^ were used to identify SCSTs and GCTs (supplementary Table 1). Up to ten controls per case were sampled among women in the MBRs with a prior pregnancy lasting longer than 22 weeks and who were alive and cancer-free at the time of the cases’ diagnosis. Controls were matched on country and the birth year of the case. Data on all the women’s pregnancies were included in the linkage to the MBRs. However, many of the women’s first pregnancies occurred before the start of the MBRs, so information was most complete for the most recent pregnancy before the case’s diagnosis/matching.

**Table 1:** Maternal and offspring characteristics of non-epithelial ovarian cancer patients and matched controls, Nordic countries, 1970–2013^a^.

Variable	Sex cord-stromal tumours		Controls		Germ cell tumours		Controls	
	*n*	%	*n*	%	*n*	%	*n*	%
Total	420	100	4041	100	345	100	2942	100
*Maternal*								
Year of birth	*n*	%	*n*	%	*n*	%	*n*	%
1925–1939	34	8.1	334	8.3	16	4.6	159	5.4
1940–1949	140	33.3	1384	34.2	46	13.3	421	14.3
1950–1959	146	34.8	1405	34.8	113	32.8	1001	34.0
1960–1969	75	17.9	702	17.4	91	26.4	752	25.6
1970–1992	25	6.0	216	5.3	79	22.9	609	20.7
Country								
Denmark	51	12.1	502	12.4	96	27.8	779	26.5
Finland	13	3.1	113	2.8	34	9.9	270	9.2
Norway	96	22.9	975	24.1	83	24.1	808	27.5
Sweden	260	61.9	2451	60.7	132	38.3	1085	36.9
Age at diagnosis/matching (years)								
<40	110	26.2	958	23.7	233	67.5	1817	61.8
40–49	143	34.0	1418	35.1	65	18.8	653	22.2
50–59	117	27.9	1169	28.9	32	9.3	330	11.2
≥60	50	11.9	496	15.2	15	4.3	142	4.8
Age at first birth (years)								
<25	131	46.3	1173	43.6	148	54.2	1123	49.5
25–29	98	34.6	907	33.7	91	33.3	780	34.4
≥30	54	19.1	613	22.8	34	12.5	366	16.1
Missing	137	–	1348	–	72	–	673	–
Age at last birth (years)								
<25	70	16.7	484	12.0	86	24.9	583	19.8
25–29	137	32.6	1235	30.6	126	36.5	1072	36.4
30–39	198	47.1	2127	52.6	123	35.7	1176	40.0
≥40	15	3.6	195	4.8	10	2.9	111	3.8
Time since first birth (years)								
<10	66	23.3	567	21.1	155	56.8	1103	48.6
10–19	84	29.7	790	29.3	74	27.1	728	32.1
20–29	89	31.4	914	33.9	32	11.7	325	14.3
≥30	44	15.5	422	15.7	12	4.4	113	5.0
Missing	137	–	1348	–	72	–	673	–
Time since last birth (years)								
<10	136	32.4	1187	29.4	234	67.8	1824	62.0
10–19	114	27.1	1269	31.4	72	20.9	705	24.0
20–29	127	30.2	1170	29.0	28	8.1	319	10.8
≥30	43	10.2	415	10.3	11	3.2	94	3.2
Number of births								
1	80	19.0	690	17.1	92	26.7	744	25.3
2	186	44.3	1781	44.1	148	42.9	1217	41.4
3	96	22.9	1007	24.9	71	20.6	607	20.6
≥4	58	13.8	563	13.9	34	9.9	374	12.7
Preeclampsia in any pregnancy								
No	386	91.9	3735	92.4	326	94.5	2778	94.4
Yes	34	8.1	306	7.6	19	5.5	164	5.6
Multiple birth in any pregnancy^b^								
No	410	97.6	3978	98.4	337	97.7	2877	97.8
Yes	10	2.4	63	1.6	8	2.3	65	2.2
Pregnancy length (weeks)^c^^,^^d^								
≤36	19	4.6	195	4.9	12	3.9	158	5.7
37–41	347	84.4	3381	85.7	268	86.2	2389	85.9
≥42	45	10.9	371	9.4	31	10.0	233	8.4
Missing	3	–	35	–	2	–	30	–
*Offspring*								
Offspring length (cm)^c^^,^^d^								
<48	31	7.6	351	8.9	17	5.5	253	9.2
48–54	357	87.7	3426	86.7	272	88.0	2361	85.4
>54	19	4.7	176	4.5	20	6.5	151	5.5
Missing	7	–	29	–	4	–	45	–
Offspring weight (g)^c^^,^^d^								
<2500	14	3.4	160	4.0	10	3.2	125	4.5
2500–4500	384	3.2	3694	92.9	293	93.9	2575	91.9
>4500	14	3.4	124	3.1	9	2.9	102	3.6
Missing	2	–	4	–	1	–	8	–

^a^Percentages are presented without including missing cases.

^b^Sex cord-stromal tumours: twin births: 10 cases/62 controls (2.4%/1.5%); triplets or greater: 0 cases/1 control (0%/0.02%). Germ cell tumours: twin births: 8 cases/63 controls (2.3%/2.1%); triplets or greater: 0 cases/2 controls (0%/0.07%).

^c^Data from last pregnancy.

^d^Excludes cases diagnosed with ovarian cancer within six months after giving birth (sex cord-stromal tumours: six cases, germ cell tumours: 32 cases).

### Exposures

We examined the following exposures: age at first and last birth; time since first and last birth; number of births at the time of matching; preeclampsia and twin/triplet pregnancy (in any pregnancy). Information on pregnancy length (in completed weeks) and offspring’s birth length and weight were recorded based on the most recent pregnancy before the case’s diagnosis to evaluate the most complete data.

### Statistical analyses

We estimated odds ratios (ORs) with 95% confidence intervals (CIs) for the association between number of births and risks of subsequent SCSTs and GCTs using conditional logistic regression models (conditioned on birth year of the case and country). Analyses of other pregnancy and neonatal characteristics were analysed in models conditioned on matching factors and additionally adjusted for number of births. Birth length/weight, adjusted for pregnancy length (as a continuous variable), were assessed as a measure of foetal growth. All factors were examined as categorical variables, and as ordinal variables to test for trend (for offspring weight, trend was tested per 500 g). In analyses of pregnancy length, and birth length/weight of offspring, women who were diagnosed with non-epithelial ovarian cancer within six months after giving birth were excluded (SCSTs: six cases, GCTs: 32 cases), to minimise the possibility that associations were influenced by preterm delivery due to cancer. We performed a sensitivity analysis in which we excluded women who had only given birth once, since their age at first and last birth is the same. We also evaluated continuous time since last birth in categories of age at last birth to better clarify the relationship between these factors. All results were tested for heterogeneity (*p-het*) of associations between countries in stratified analyses, using a likelihood ratio test. R version 3.3.2 was used for all analyses.^24^

## Results

We identified 420 cases of SCSTs in parous women, with a median age of 47 years (range 21–71) at diagnosis and 345 cases of GCTs in parous women, with a median age of 37 years (range 18–79). Maternal characteristics and data on the offspring from the most recent pregnancy, stratified by case/control status, are presented in Table 1.

### Risk factors for sex cord-stromal tumours

Higher age at last birth was associated with decreased risk of SCSTs [i.e. last birth at age ≥40, versus before 25 years: OR 0.48 (95% CI 0.23–0.98); per year increasing age: OR 0.97 (95% CI 0.94–0.99)]. A similar trend was seen with higher age at first birth [first birth at age ≥30, versus before 25 years: OR 0.70 (95% CI 0.47–1.05); per year increasing age: OR 0.97 (95% CI 0.94–1.00)]. SCST risk decreased gradually with higher age at first or last birth (Table 2). A recent childbirth (shorter time since first and last birth) was associated with decreased risk (Table 2). Increasing number of births was not associated with risk of SCSTs, nor were any of the other investigated factors. All results are presented adjusted for number of births, but unadjusted results were very similar (supplementary Table 2).

**Table 2:** Risk of non-epithelial ovarian cancer by subtype for pregnancy, perinatal and birth characteristics^a^.

	Sex cord-stromal tumours		Germ cell tumours	
Cases/controls	420/4041 (54.9%)		345/2942 (45.1%)	
Mean/median age at diagnosis (range)	48/47 (21–71)		39/37 (18–79)	
	OR	95% CI	OR	95% CI
Age at first birth (years)				
<25	1.00	Ref	1.00	Ref
25–29	0.91	0.67–1.24	0.98	0.71–1.34
≥30	0.70	0.47–1.05	0.93	0.59–1.47
Per year age	0.97	0.94–1.00	1.01	0.98–1.05
Age at last birth (years)				
<25	1.00	Ref	1.00	Ref
25–29	0.77	0.55–1.08	1.03	0.72–1.46
30–39	0.64	0.45–0.90	1.01	0.68–1.49
≥40	0.48	0.23–0.98	0.82	0.31–2.16
Per year age	0.97	0.94–0.99	1.01	0.98–1.04
Time since first birth (years)				
<10	0.53	0.23–1.19	1.20	0.36–3.96
10–19	0.69	0.35–1.35	1.03	0.34–3.15
20–29	0.80	0.47–1.38	0.96	0.37–2.53
≥30	1.00	Ref	1.00	Ref
Per year	1.03	1.00–1.07	0.99	0.95–1.02
Time since last birth (years)				
<10	0.78	0.40–1.53	0.81	0.23–2.78
10–19	0.70	0.39–1.25	0.76	0.24–2.48
20–29	0.97	0.60–1.56	0.61	0.21–1.81
≥30	1.00	Ref	1.00	Ref
Per year	1.04	1.01–1.06	0.99	0.96–1.02
Number of births				
1	1.00	Ref	1.00	Ref
2	0.97	0.72–1.29	1.19	0.88–1.60
3	0.89	0.64–1.24	1.19	0.83–1.70
≥4	0.96	0.65–1.41	0.95	0.60–1.50
Per birth	0.96	0.87–1.05	0.99	0.90–1.10
Preeclampsia in any pregnancy				
No	1.00	Ref	1.00	Ref
Yes	1.09	0.75–1.58	1.08	0.65–1.78
Multiple birth in any pregnancy				
No	1.00	Ref	1.00	Ref
Yes	1.49	0.75–2.98	1.16	0.55–2.47
Pregnancy length (weeks)^b^^,^^c^				
≤36	0.91	0.55–1.49	0.72	0.39–1.33
37–41	1.00	Ref	1.00	Ref
≥42	1.18	0.84–1.64	1.23	0.82–1.85
Per week	1.03	0.97–1.09	1.07	1.00–1.14
Offspring length (cm)^b^^,^^c^^,^^d^				
<48	0.90	0.59–1.37	0.64	0.37–1.13
48–54	1.00	Ref	1.00	Ref
>54	1.02	0.62–1.68	1.11	0.67–1.83
Per cm	1.04	0.99–1.09	1.02	0.96–1.07
Offspring weight (g)^b^^,^^c^^,^^d^				
<2500	0.79	0.41–1.50 88	0.98	0.46–2.07
2500–4500	1.00	Ref	1.00	Ref
>4500	1.13	0.64–1.98	0.78	0.38–1.58
Per 500 g	1.05	0.94–1.15	1.06	0.93–1.18

^a^Odds ratio (OR) and 95% confidence intervals (CIs) from conditional logistic regression models, conditioned on birth year (of the case) and country, adjusted for number of births.

^b^Data from last pregnancy.

^c^Excludes cases diagnosed with ovarian cancer within six months after giving birth (sex cord-stromal tumours: six cases, germ cell tumours: 32 cases).

^d^Adjusted for pregnancy length (last pregnancy) as a continuous variable.

### Risk factors for germ cell tumours

Age at birth was not associated with GCTs [i.e. last birth at age 30–39 versus before 25 years: OR 1.01 (95% CI 0.68–1.49)], nor were time since birth, number of births [per birth: OR 0.99 (95% CI 0.90–1.10)], or any of the other investigated factors.

### Sensitivity analyses

We performed a sensitivity analysis for SCSTs in which we excluded women who had only given birth once, but the results were not altered [i.e. last birth at age 30–39, versus before 25 years: OR 0.61 (95% CI 0.41–0.90)]. The proportion SCSTs and GCTs differed between the Nordic countries, with a higher percentage of SCSTs in Norway and Sweden and a higher percentage of GCTs in Denmark and Finland (Table 1). However, when we stratified our analyses and tested for heterogeneity of associations among countries, the ORs between the pregnancy-related factors and SCSTs and GCTs did not differ (supplementary Table 3), suggesting there were no major differences in coding between the countries.

We evaluated time since last birth in categories of age at last birth and vice versa (supplementary Table 4). The pattern was consistent across both, with reductions in risk for the recent births (whether categorised by ≥40 age at last birth, or by <10 years since last birth).

## Discussion

In this large study of non-epithelial ovarian cancer, we found that both higher age at last birth and shorter time since last birth were associated with decreased risk of SCSTs, but not with risk of GCTs. There was no association between number of births or any of the other investigated maternal or offspring factors and risk of SCSTs or GCTs.

### Sex cord-stromal tumours

The evidence of a relation between age at birth and risk of SCSTs have so far been limited by few, and often small studies (summarised in supplementary Table 5). In the largest study to date, our findings of associations between higher age at last birth and shorter time since last birth and subsequent decreased risk of SCSTs, are, however, consistent with two smaller studies. A small Mexican case-control study,^7^ (based on three cases that were over 28 years of age at their last birth), and a study of Swedish women born 1925–1960 who were diagnosed with ovarian cancer during 1958–1984 (hence partly overlapping with this study), showed higher age at first birth tended to be associated with a decreased risk of SCSTs.^10^ In contrast, an older American study^6^ published in 1992 with 45 SCSTs found an increased risk with higher age at first birth and SCSTs. In this study, nulliparous women were included, and the results were based on only seven SCST cases that were over 29 years of age at their first birth, and the effect of age at last birth was not evaluated, restricting the generalisability of the findings.

Our finding with a lack of association between number of births and SCSTs is consistent with three previous studies,^6,8,9^ although one of them found an association between nulliparity and SCSTs,^9^ which we did not analyse. In addition, two other studies^7,10^ found an association between increasing number of births and decreased risk of SCSTs: The study, by Adami et al.,^10^ was comprised of data from an earlier calendar period when diagnostic criteria might not have been as well defined as today resulting in misclassified epithelial ovarian cancer cases. The second study, by Sanches-Zomorano et al.,^7^ was based on only 10 cases. All studies except Adami et al. included nulliparous women in their analyses. In light of our findings, and review of other studies, the impact of number of births on risk of SCSTs seems limited.

To our knowledge, prior investigations on preeclampsia, multiple birth and pregnancy length, and birth characteristics (offspring length and weight) on risk of non-epithelial ovarian cancer have not been performed. Our study does not, however, support associations between these characteristics and subsequent risk of SCSTs in the mother.

In summary, data from our large study suggest that older age at last birth, and shorter time since last birth affect risk of SCSTs. Results from previous studies are somewhat conflicting (summarised in supplementary Table 5). A hormonal influence in this subgroup is suggested by data from a 2011 Finnish study showing a positive association between higher levels of testosterone, androstenedione and 17-OH-progesterone concentrations during pregnancy and risk of SCSTs.^11^ However; effects of 17-OH-progesterone might differ from those of progesterone, and timing of hormonal exposure in pregnancy may be important. Another Finnish study^25^ measured hormone levels during the first trimester in women with singleton pregnancies, and found that maternal age was associated with sex steroids concentrations, with higher progesterone concentrations and lower androgen and oestradiol in women >30 years of age. An American study from 2008,^26^ found similar results with a positive association between higher maternal age and levels of progesterone, and inverse associations with androgen and oestrogen. This could be a possible explanation for the decrease in risk of SCSTs with higher age at last birth, with progesterone levels associated with a decrease in risk and androgens with an increase in risk. A risk decrease with increasing progesterone levels has been raised as a potential protective factor for epithelial ovarian cancer through cell clearance of pre-malignant cells (reviewed in ref. ^27^) and indicates similar mechanisms for SCSTs and epithelial ovarian cancer. The increased risk with increasing androgen and oestradiol concentrations is generally consistent with SCSTs being rare before puberty, when hormone levels are low. These hormones are likely needed for tumour development or progression.

### Germ cell tumours

Our finding of a lack of association between age at birth and GCTs is consistent with two previous studies.^7,10^ A Norwegian cohort study, including 71 cases of GCTs diagnosed 1960–1991 (hence partly overlapping with our study),^8^ found an increased risk of GCTs with high-aged childbirths. This result was, however, based on only 40 parous women. As in previous studies,^6–8,10^ we saw no association between number of births and GCTs. Neither did we see any associations between the analysed pregnancy and birth characteristics and subsequent risk of GCTs in the mother.

To summarise, a woman’s reproductive history does not seem to influence her risk of GCTs. Gonadal dysgenesis is associated with risk of GCTs,^28,29^ but not SCSTs. This suggests that development of GCTs is more genetically controlled, given that gonadal dysgenesis is secondary to chromosomal anomalies or mutations in genes related to the urogenital ridge or sex differentiation.^28^ This genetic component may also explain why there are no specific reproductive risk factors for GCTs, as opposed to SCSTs. Changes in hormone levels during puberty might play a role, while the greater alterations during pregnancy have a more limited ability to affect risk because affected individuals would need to be very young at childbirth.

### Strength and limitations

This population-based study is based on the largest number of SCSTs and GCTs reported to date. Despite the comparably larger sample size, statistical power was still limited, and confidence intervals were often wide suggesting that even larger studies are needed to reach firmer conclusions about associations that are likely modest. The cases numbers also limited our ability to separate the effect of the correlated factors of higher age at last birth and shorter time since last birth.

Among the strengths of this study is the almost complete information on the studied perinatal factors, based on mandatory reporting to birth registries which avoids recall bias. The nationwide cancer registries in the four Nordic countries^22^ enabled identification of cancer cases in a similar and standardised manner. However, the proportions SCSTs and GCTs differed between the Nordic countries, which could imply differences in coding, genetics, or other risk factors, although there was no evidence of heterogeneity in the ORs by country.

While there are few, if any, established risk factors for non-epithelial ovarian cancer, it is unclear if the current study was missing information on important confounding factors, such as obesity, breast-feeding and oral contraceptive use. Oral contraceptives are associated with decreased risk of epithelial, and possible non-epithelial, ovarian cancer,^6,30,31^ but since data are scarce, their impact on SCSTs and GCTs remains uncertain. We also lacked information on oophorectomy/salpingectomy or hysterectomy, and thus could not exclude controls that had undergone gynaecological surgery. However, since the prevalence of women having gone through a oophorectomy/hysterectomy in the Nordic countries is low (200 per 100,000 women), and probably even lower in this young cohort, it is unlikely to have had a major effect on our risk estimates.^32^ Because our aim was to study the impact of pregnancy-related factors on risk of SCSTs and GCTs, we only included parous women in our study and results are thus generalisable to parous women only. This resulted in a higher mean age of GCT cases than in the general population and our inability to investigate the influence of early pregnancy loss (prior to week 22).

## Conclusions

In this population-based case-control study, including 420 cases of SCSTs and 345 cases of GCTs among parous women, higher age at last birth, and shorter time since last birth, were both associated with a decreased risk of SCSTs, similar to results for epithelial ovarian cancer. Thus, it is possible that hormonal mechanisms or other mechanisms related to this aspect of reproductive history influence the development of both SCSTs (but not GCTs) and epithelial ovarian cancer. Number of births was not associated with the risk of SCSTs or GCTs. We found no associations with any of the investigated pregnancy-related factors and GCTs, suggesting that reproductive factors may have limited impact on risk of these uncommon tumours.

## Supplementary information

Supplementary files

## Data Availability

The data in the current manuscript are results of linkages of several nationwide registers as described in the method section. Procedures for accessing the data will be available from the corresponding author.
