# Supplementary material for: Pregnancy-related risk factors for sex cord-stromal tumours and germ cell tumours in parous women: a registry-based study
Source: Br J Cancer. 2020 Apr 27;123(1):161–6. doi: 10.1038/s41416-020-0849-z (PMC7340776; doi:10.1038/s41416-020-0849-z)
Supplement: Supplementary file 1 — Supplementary files [file 41416_2020_849_MOESM1_ESM.docx]

**Supplementary Table 1.**

**ICD codes used to identify cases^23^**

|  | **ICD-O/2, ICD-O/3^1^** | **ICD-7** **175.0 + WHO/HS/CAN/C24.1**^1^ | **ICD-7^2^** |
| --- | --- | --- | --- |
| Germ cell tumors | 8070, 8240, 8243, 8410, 8720, 9060, 9070, 9071, 9072, 9073, 9080, 9084, 9085, 9090, 9091, 9100, 9363, 9391,  9440, 9473, 9501 | 066, 086, 826 | 1753, 3750 |
| Sex cord-stromal tumors | 8590, 8593, 8600, 8601, 8602, 8610, 8620, 8622, 8623, 8631, 8632, 8633, 8634, 8640, 8650, 8660, 8670, 8810 | 056, 706 | 4750 |

^1^Norway, Denmark and Finland: ICD-O-3 morphology codes. Sweden: ICD-O/2 and ICD-O/3 from 1993, ICD-7 175.0 + WHO/HS/CAN/C24.1 until 1993. Denmark: ICD-7 codes were used prior to 1978: ^2^http://www.dst.dk/extranet/staticsites/TIMES3/html/6b12c6c3-b1e2-490b-b031-76b0294132db.htm.

**Supplementary Table 2.**

**Risk of non-epithelial ovarian cancer by subtype for pregnancy, perinatal and birth characteristics, unadjusted results***

|  | | | **Sex cord-stromal tumors** | | **Germ cell tumors** | |
| --- | --- | --- | --- | --- | --- | --- |
| **Cases/ controls** | | | **420/4041 (54.9%)** | | **345/2942 (45.1%)** | |
| Mean/ median age at diagnosis (range)  (range) | | | 48/ 47 (21-71) | | 39/ 37 (18-79) | |
|  | | | **OR** | **95% CI** | **OR** | **95% CI** |
| **Age at first birth (years)** | | |  |  |  |  |
| <25 | | | 1.00 | Ref | 1.00 | Ref |
| 25-29 | | | 0.94 | 0.70-1.28 | 0.96 | 0.71-1.31 |
| ≥30 | | | 0.76 | 0.51-1.12 | 0.91 | 0.59-1.39 |
| Per year age | | | 0.98 | 0.95-1.01 | 1.01 | 0.97-1.04 |
| **Age at last birth (years)** | | |  |  |  |  |
| <25 | | | 1.00 | Ref | 1.00 | Ref |
| 25-29 | | | 0.77 | 0.55-1.07 | 1.03 | 0.72-1.46 |
| 30-39 | | | **0.63** | **0.45-0.89** | 1.00 | 0.68-1.48 |
| ≥40 | | | **0.47** | **0.24-0.95** | 0.82 | 0.32-2.11 |
| Per year age | | | **0.97** | **0.94-0.99** | 1.01 | 0.98-1.04 |
| **Time since first birth (years)** | | |  |  |  |  |
| <10 | | | 0.60 | 0.27-1.32 | 1.09 | 0.34-3.50 |
| 10-19 | | | 0.74 | 0.38-1.43 | 0.97 | 0.32-2.95 |
| 20-29 | | | 0.82 | 0.48-1.41 | 0.94 | 0.36-2.46 |
| ≥30 | | | 1.00 | Ref | 1.00 | Ref |
| Per year | | | 1.02 | 0.99-1.06 | 0.99 | 0.96-1.03 |
| **Time since last birth (years)** | | |  |  |  |  |
| <10 | | | 0.75 | 0.39-1.43 | 0.79 | 0.23-2.70 |
| 10-19 | | | 0.68 | 0.38-1.20 | 0.75 | 0.23-2.44 |
| 20-29 | | | 0.96 | 0.60-1.54 | 0.61 | 0.20-1.80 |
| ≥30 | | | 1.00 | Ref | 1.00 | Ref |
| Per year | | | **1.04** | **1.01-1.06** | 0.99 | 0.96-1.02 |
| **Number of births** | | |  |  |  |  |
| 1 | | | 1.00 | Ref | 1.00 | Ref |
| 2 | | | 0.97 | 0.72-1.29 | 1.19 | 0.88-1.60 |
| 3 | | | 0.89 | 0.64-1.24 | 1.19 | 0.83-1.70 |
| ≥4 | | | 0.96 | 0.65-1.41 | 0.95 | 0.60-1.50 |
| Per birth | | | 0.96 | 0.87-1.05 | 0.99 | 0.90-1.10 |
| **Preeclampsia in any pregnancy** | | |  |  |  |  |
| No | | | 1.00 | Ref | 1.00 | Ref |
| Yes | | | 1.09 | 0.75-1.58 | 1.07 | 0.65-1.77 |
| **Multiple birth in any pregnancy** | | |  |  |  |  |
| No | | | 1.00 | Ref | 1.00 | Ref |
| Yes | | | 1.44 | 0.72-2.86 | 1.15 | 0.55-2.44 |
| **Pregnancy length (weeks)^1,2^** | | |  |  |  |  |
| ≤36 | | | 0.91 | 0.55-1.49 | 0.72 | 0.39-1.33 |
| 37-41 | | | 1.00 | Ref | 1.00 | Ref |
| ≥42 | | | 1.18 | 0.84-1.64 | 1.23 | 0.82-1.85 |
| Per week | | | 1.03 | 0.97-1.09 | 1.07 | 1.00-1.14 |
| **Offspring length (cm)^1-3^** | |  |  |  |  |  |
|  | <48 | | 0.90 | 0.59-1.37 | 0.64 | 0.37-1.13 |
|  | 48-54 | | 1.00 | Ref | 1.00 | Ref |
|  | >54 | | 1.01 | 0.62-1.67 | 1.11 | 0.67-1.82 |
|  | Per cm | | 1.04 | 0.99-1.09 | 1.02 | 0.96-1.07 |
| **Offspring weight (g) ^1-3^** | | |  |  |  |  |
| <2500 | | | 0.79 | 0.41-1.50  88 | 0.98 | 0.46-2.07 |
| 2500-4500 | | | 1.00 | Ref | 1.00 | Ref |
| >4500 | | | 1.11 | 0.63-1.96 | 0.78 | 0.38-1.57 |
| Per 500 g | | | 1.04 | 0.94-1.15 | 1.06 | 0.93-1.18 |

*Odds ratio (OR) and 95% confidence intervals (CIs) from conditional logistic regression models, conditioned on birth year (of the case) and country.

^1^Data from last pregnancy.

^2^Excludes cases diagnosed with ovarian cancer within six months after giving birth (sex cord-stromal tumors: 6 cases, germ cell tumors: 32 cases).

^3^Adjusted for pregnancy length (last pregnancy) as a continuous variable.

**Supplementary Table 3.**

**Test for heterogeneity between Denmark, Finland, Norway and Sweden**

**Sex cord-stromal tumors Germ cell tumors**

|  | | *p-het* | *p-het* |
| --- | --- | --- | --- |
| **Number of births** | | 0.61 | 0.96 |
|  | |  |  |
| **Age at first birth** | | 0.98 | 0.48 |
|  | |  |  |
| **Age at last birth** | | 0.26 | 0.68 |
|  | |  |  |
| **Time since first birth** | | 0.89 | 0.90 |
|  | |  |  |
| **Time since last birth** | | 0.18 | 0.99 |
|  | |  |  |
| **Preeclampsia in any pregnancy** | | 0.54 | 0.49 |
|  | |  |  |
| **Multiple birth in any pregnancy** | | 0.11 | 0.86 |
|  | |  |  |
| **Pregnancy length** | | 0.67 | 0.14 |
|  | |  |  |
| **Offspring length** |  | 0.22 | 0.49 |
|  |  |  |  |
| **Offspring weight** | | 0.60 | 0.80 |
|  | |  |  |

**Supplepementary Table 4.**

**Risk of sex cord-stromal tumors by time sinced last birth, stratified on number of births, conditional logistic regression.**

| **Age at last birth** | **<25** | | **25-29** | | **30-39** | | ≥**40** | |
| --- | --- | --- | --- | --- | --- | --- | --- | --- |
|  | **OR** | **95% CI** | **OR** | **95% CI** | **OR** | **95% CI** | **OR** | **95% CI** |
| **Number of cases/controls** | 70/484 | | 137/1235 | | 198/2127 | | 15/195 | |
| **Time since last birth (years)** |  |  |  |  |  |  |  |  |
| Per year | 1.19 | 0.97-1.46 | 1.04 | 0.90-1.20 | 1.01 | 0.95-1.08 | 0.77 | 0.46-1.30 |

**Risk of sex cord-stromal tumors by age at last birth, stratified on time since last birth, conditional logistic regression.**

| **Time since last birth** | **<10** | | **10-19** | | **20-29** | | **≥30** | |
| --- | --- | --- | --- | --- | --- | --- | --- | --- |
|  | **OR** | **95% CI** | **OR** | **95% CI** | **OR** | **95% CI** | **OR** | **95% CI** |
| **Number of cases/controls** | 136/1187 | | 114/1269 | | 127/1170 | | 43/415 | |
| **Age at last birth (years)** |  |  |  |  |  |  |  |  |
| Per year | 0.90 | 0.83-0.97 | 0.94 | 0.86-1.02 | 0.96 | 0.88-1.04 | 1.03 | 0.88-1.20 |

Supplementary Table 5

| **Study (year)** | **Factors studied** | **Cases (n)** | **Study design** | **Study population, geographic area** | **Variables adjusted for** | **Study period** | **Median age in years (range)** | **Results** |
| --- | --- | --- | --- | --- | --- | --- | --- | --- |
| Sköld  (2019)  (Current investigation) | - Number of births,  - Age at first and last birth,  - Time since first and last birth,  - Preeclampsia,  - Twin/ triplet pregnancy,  - Pregnancy length,  - Offspring’s birth length and weight | SCSTs: 420; GCTs: 345 | Case-control study | Nordic countries | Number of births | 1970-2013 | SCSTs: 47 (21-71);  GCTs: 37 (18-79) | **Nulliparity:** Not investigated.  **Number of births:** SCSTs: no ass. GCTs: no ass.  **Age at births:** SCSTs: decreased risk with high-age childbirths. GCTs: no ass.  **i.e. High-age at childbirth protects against SCSTs ↓** |
| Boyce (2009) | - Race,  - Obesity,  - Number of births,  - Smoking,  - Family history | SCSTs: 72  (granulosa  cell tumors) | Case-control study | USA (Brigham and Women's Hospital  and the Massachusetts and New Hampshire Statewide Cancer Registries) | Race and age | 1988-2008 | 15-80 | **Nulliparity:** SCSTs: increased risk.  **Number of births:** SCSTs: No ass.  **Age at births:** Not investigated.  **i.e. Age at childbirth not investigated** |
| Sanchez-Zamorano (2003) | - Number of pregnancies,  - Age at first and last birth,  - Time since last birth,  - Breastfeeding,  - Weight change in past 10 years and 6 months,  - Menopausal status,  - Oral contraceptive use | SCSTs: 10;  GCTs: 18 | Case-control study | Mexico (a reference hospital in southern Mexico City) | Hormonal contraceptives and parity | 1995-1997 | SCSTs: 49 (18-76);  GCTs: 23 (12-65) | **Nulliparity:** SCSTs: increased risk. GCTs: no ass.  **Number of births:** SCSTs: decreased risk. GCTs: no ass.  **Age at births:** SCSTs: decreased risk with high-age childbirths. GCTs: no ass.  **i.e. High-age at childbirth protects against SCSTs ↓** |
| Albrektsen (1997) | - Pregnancy,  - Age at birth | SCSTs: 41;  GCTs: 71. | Cohort study | Norway | Number of births | 1960-1991 | 20-56 | **Nulliparity:**  SCSTs: no ass. GCTs: no ass.  **Number of births:** SCSTs: no ass. GCTs: no ass.  **Age at births:** SCSTs: no ass. GCTs: increased risk with high-age childbirths.  **i.e. Age at childbirth not associated with SCSTs ↔** |
| Adami (1994) | - Number of births,  - Age at first birth | SCSTs: 330;  GCTs: 149 | Case-control study | Sweden | Number of births, age at first birth. | 1958-1984 | Unknown | **Nulliparity:** Not investigated.  **Number of births:** SCSTs: decreased risk. GCTs: no ass.  **Age at births:** SCSTs: trend to decreased risk with high-age childbirths. GCTs: no ass.  **i.e. Age at childbirth potentially associated with SCSTs ↓?** |
| Horn-Ross (1992) | - Age at menarche,  - Oral contraceptives,  - Parity,  - Age at first term birth,  - Duration of lactation,  - Body mass index,  - Alcohol use,  - Estrogen replacement use | SCSTs: 45;  GCTs: 38 | Case-control study | USA (Collaborative Ovarian Cancer Group) | Age, study, year of birth, oral contraceptives, parity | 1975-1982 | Unknown | **Nulliparity:**  SCSTs: no ass. GCTs: no ass.  **Number of births:** SCSTs: no ass. GCTs: no ass.  **Age at births:** SCSTs: increased risk with high-age childbirths. GCTs: not evaluated.  **i.e. High-age at childbirth increases risk of SCSTs** **↑** |
